# Bilateral neuro-retinitis following chick embryo cell anti-rabies vaccination – a case report

**DOI:** 10.1186/1471-2415-5-20

**Published:** 2005-08-17

**Authors:** Rohit Saxena, Harinder Singh Sethi, Harminder Kumar Rai, Vimla Menon

**Affiliations:** 1 Dr. R. P. Centre for Ophthalmic Sciences, All India Institute of Medical Sciences, New Delhi 110029, India

## Abstract

**Background:**

The Optic nerve is rarely involved after sheep brain anti-rabies vaccination in the form of retrobulbar neuritis or papillitis. Bilateral neuroretinitis after chick embryo cell antirabies vaccination has not been reported.

**Case presentation:**

We report the case of a 56 year old male who developed bilateral neuro-retinitis following three injections of antirabies vaccine prepared from the chick embryo.

**Conclusion:**

The chick embryo cell antirabies vaccine can cause bilateral neuroretinits which has not been reported previously.

## Background

The Optic nerve is rarely involved after sheep brain anti-rabies vaccination. Neurological complications are usually seen with sheep brain vaccines but can be rarely seen after chick embryo cell vaccines [1]. The main cause in such cases is presumed to be the antigenic cerebral tissue used in the preparation of sheep brain vaccine [2-5]. We report the case of a 56 year old male who developed bilateral neuro-retinitis following three injections of antirabies vaccine prepared from the chick embryo. Retrobular neuritis and papillitis following sheep brain antirabies vaccine have been reported [6-11]. The present report describes a case with bilateral neuroretinitis after chick embryo antirabies vaccine, which to best of our knowledge has not been reported earlier.

## Case presentation

A 56 year old male presented with complaint of acute onset painless diminution of vision in the right eye of 3 days duration followed by similar complaint in left eye after 1 day which deteriorated over the next 2 days. This was associated with headache and pain on ocular movements. There was a history of being bitten by a stray dog 8 days before the visual symptoms, and the patient had received three injections of chick embryo cell anti-rabies vaccine (Rabipur, Hoeshst Marion Roussel) on day 0, 3 and 7 after the dog bite. He developed these symptoms after the third injection. There was no history of any other ocular or systemic problems.

**Figure 1:**
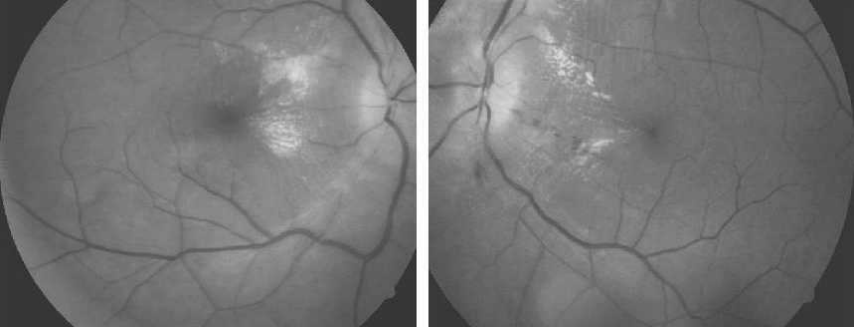
Fundus photograph of right and left eye depicting bilateral neuroretinitis.

The general physical examination was within normal limits. The best-corrected visual acuity was 6/60 in the right and 6/24 in the left eye. IOP was 16 mm of Hg by applanation tonometry. Anterior segment examination using slitlamp was normal. Fundoscopy using + 90 D revealed hyperemic optic disc with blurred margins and disc edema. Vessels were tortuous and macular star was present in the nasal part of macula of both the eyes (Fig 1). Pupils were sluggishly reacting in both the eyes. Goldmann perimetry revealed centrocaecal scotoma in both eyes which was more dense in the right eye as compared to the left.

The routine blood investigations were all within normal limits. The aerobic and aerobic cultures were sterile. The VDRL test and indirect haemagglutination test for syphillis and toxoplasmosis were normal. Serology for bartonella was negative. Mantoux test showed a reading of 8 mm. and the chest X-ray was normal. Magnetic resonance imaging (MRI) brain and optic nerves revealed slight signal enhancement of both the optic nerves with slight increase in optic nerve thickness on axial T2-weighted magnetic resonance imaging. No other significant abnormality was seen in MRI of brain.

A diagnosis of bilateral neuroretinitis was made. As the dog which had bitten the patient was kept under observation and was alive after one week, further injections of antirabies vaccine were stopped. The patient was given three doses of pulse dexamethasone 100 mg in 150 ml of 5% dextrose. The visual acuity improved to 6/6 in both the eyes over the next two weeks. The disc oedema start resolving within one week but macular exudates took 4 months to resolve.

## Discussion

Neuroretinitis is an acute swelling of the optic disc associated with hard exudates or edema around the macula. They are frequently associated with infectious diseases but may also occur without any apparent cause (Leber's Idiopathic Stellate neuroretinitis). Infectious causes reported in literature include syphilis, toxoplasmosis, toxocariosis, Lyme's disease and Cat scratch disease. Antecedent viral infection especially herpes simplex virus (HSV), Hepatitis B and mumps have also been reported. It is a self limiting disease with a tendency to recover spontaneously. No treatment is also an option as the condition recovers spontaneously but treatment may speed the recovery and shorten the course of the disease. The cases with underlying bacterial etiology require the specific antibiotic therapy [12,13].

Neurologic complications after sheep brain anti rabies vaccination, although rare can be very serious. These include encephalitis, myelitis, encephalomyelitis and paralysis of various cranial nerves[2]. Optic nerve involvement is not a frequently reported complication and is usually a part of diffuse encephalomyelitis. Antigenecity of the sheep brain is generally held responsible for the neuroparalytic complications [1,2]. The onset of symptoms may vary from 1 to 3 weeks to as much as 100 days after vaccination. Immune complex mediated vascular injury jeopardizing the blood-brain barrier has been proposed as a possible mechanism, which leads to lymphocyte mediated demyelinaton and inflammation [4]. In all previously reported cases of optic neuritis following antirabies vaccination, sheep brain vaccine had been used. However our present case had received chick embryo vaccine. Chakravarty has reported a single case of neurologic illness simultating Guillain Barre syndrome after post-exposure prophylaxis with purified chick embryo cell anti-rabies vaccine [1]. Apart from this, no published reports are available regarding the neurological complication following purified chick embryo cell vaccination (although manufacturer's information on their websites does mention the risk of neurological complications with some chick embryo vaccines). This case was also unusual in the sense that patient has neuroretinits as compared to previously reported cases which have retrobulbar neuritis or papillitis [6-11]. Although good recovery is reported spontaneously, pulse steroids speed up the recovery. Our patient had good visual recovery after three doses of pulse steroids. As brain tissue and perhaps chick embryo tissue (as in our case) is thought to be the cause of the neuroparalytic complications, it is suggested that recombinant vaccines may offer a solution to this problem.

## Conclusion

The chick embryo antirabies vaccine can cause bilateral neuroretinits which has not been reported previously.

## Competing interests

The author(s) declare that they have no competing interests.

## Authors' contributions

RS : Helped in making the clinical diagnosis and management of the case.

HSS : Maintained the follow-up record, and prepared the manuscript.

HKR : Performed the literature search and helped in documentation of case

VM : Helped in making the clinical diagnosis and management of the case and supervised the case report.

All authors read and approved the final manuscript.

## Pre-publication history

The pre-publication history for this paper can be accessed here:
